# Virus-Mediated Alterations in miRNA Factors and Degradation of Viral miRNAs by MCPIP1

**DOI:** 10.1371/journal.pbio.2000998

**Published:** 2016-11-28

**Authors:** Christine Happel, Dhivya Ramalingam, Joseph M. Ziegelbauer

**Affiliations:** HIV and AIDS Malignancy Branch, National Cancer Institute, National Institutes of Health, Bethesda, Maryland, United States of America; University of Wisconsin-Madison, United States of America

## Abstract

Kaposi’s sarcoma-associated herpesvirus (KSHV), the causative agent of Kaposi’s sarcoma, encodes 25 mature viral miRNAs. MCP-1-induced protein-1 (MCPIP1), a critical regulator of immune homeostasis, has been shown to suppress miRNA biosynthesis via cleavage of precursor miRNAs through its RNase domain. We demonstrate that MCPIP1 can directly cleave KSHV and EBV precursor miRNAs and that MCPIP1 expression is repressed following de novo KSHV infection. In addition, repression with siRNAs to MCPIP1 in KSHV-infected cells increased IL-6 and KSHV miRNA expression, supporting a role for MCPIP1 in IL-6 and KSHV miRNA regulation. We also provide evidence that KSHV miRNAs repress MCPIP1 expression by targeting the 3’UTR of MCPIP1. Conversely, expression of essential miRNA biogenesis components Dicer and TRBP is increased following latent KSHV infection. We propose that KSHV infection inhibits a negative regulator of miRNA biogenesis (MCPIP1) and up-regulates critical miRNA processing components to evade host mechanisms that inhibit expression of viral miRNAs. KSHV-mediated alterations in miRNA biogenesis represent a novel mechanism by which KSHV interacts with its host and a new mechanism for the regulation of viral miRNA expression.

## Author Summary

A subset of viruses, including Kaposi’s sarcoma-associated herpesvirus (KSHV), encode microRNAs within their own genomes. We propose that the human host may possess strategies to repress viral microRNA biogenesis as an antiviral strategy by taking advantage of unique temporal features of viral microRNA biogenesis upon infection. We describe a human protein that can degrade microRNA precursor molecules from two different viruses. In this report, we identified strategies by which KSHV can evade this antiviral host response through alterations in the expression of its microRNA biogenesis factors. We found an inverse relationship between key microRNA biogenesis factors and inhibitors of microRNA stability after KSHV infection. KSHV-mediated alterations in microRNA biogenesis factor expression represent a novel mechanism by which KSHV interacts with its host and a mechanism for the regulation of viral microRNA expression.

## Introduction

Kaposi’s sarcoma-associated herpesvirus (KSHV; HHV-8), is a γ-herpesvirus that is the causative agent of Kaposi’s sarcoma (KS) in endothelial cells and two rare lymphoproliferative disorders, primary effusion lymphoma (PEL) and multicentric Castleman’s disease (MCD). KS is an AIDS-defining malignancy and the most frequent cancer in many sub-Saharan countries [1–3]. It should be noted that the majority of infected cells in KS lesions are latently infected [4].

MicroRNAs (miRNAs) are small (21–23 nt) noncoding RNAs that regulate gene expression post-transcriptionally through translational repression and/or mRNA degradation. The KSHV genome encodes 12 pre-miRNAs that give rise to at least 18 mature viral miRNAs. In KSHV-associated cancers, a single KSHV miRNA can account for as much as 28% of all human and viral mature miRNAs within a cell and function to regulate host gene expression to promote the latent viral persistence, immune evasion, and tumor progression [5,6].

Regardless of species of origin, most miRNAs are expressed and processed in similar fashion. Primary microRNAs (pri-miRNAs) are cleaved by the Drosha/DGCR8 complex to form hairpin precursor miRNAs (pre-miRNAs). Pre-miRNAs are then exported into the cytoplasm where they are further cleaved by the RNase III enzyme, Dicer. During processing and loading of mature miRNAs, Dicer collaborates with members of the Argonaute protein family (AGO1-4) and HIV-1 TAR-RNA Binding Protein (TARBP2 or TRBP) [7,8]. This intermediate complex is termed the miRNA RISC loading complex (miRLC) [9]. One strand representing the ~22 nt mature miRNA can then be loaded into the RNA-induced silencing complex (RISC), while Dicer and TRBP are released [9]. The miRNA-induced silencing complex (miRISC) can then interact with target mRNAs [10]. Due to the imperfect complementarity with which miRNAs bind to target sequence mRNAs, target prediction remains difficult.

MCP-1-induced protein-1 (MCPIP1 or Regnase-1) is part of the CCCH-zinc finger protein family that includes MCPIP1, 2, 3, and 4 and is encoded by four genes *Zc3h12a*, *Zc3h12b*, *Zc3h12c*, and *Zc3h12d*, respectively [11]. MCPIP1 is expressed in most tissues and can be induced in response to treatment with a number of proinflammatory molecules such as interleukin-1β (IL-1β), monocyte chemoattractant protein-1 (MCP-1) and tumor necrosis factor α (TNFα) [12,13]. MCPIP1 has been described as a transcription factor and a RNase due to its PIN-like nuclease domain [14], which acts as a negative regulator of inflammation due to its ability to degrade the mRNA transcripts of the proinflammatory cytokines IL-1, IL-6, and IL-12p40 [12,15]. Recently, MCPIP1 was suggested to be a broad suppressor of the miRNA biogenesis pathway by cleaving the terminal loops of pre-miRNAs leading to destabilization of pre-miRNAs and, ultimately, degradation [16]. At the time of infection, most human miRNAs exist in the mature miRNA form in the RISC complex. However, incoming viral miRNAs are distinct because these viral miRNAs start at the beginning of the biogenesis pathway, which makes them more sensitive to MCPIP1 since it cleaves pre-miRNAs and not mature miRNAs.

In this report, we demonstrate that MCPIP1 can directly cleave human and viral pre-miRNAs and reduce mature miRNA levels. We identify MCPIP1 as a down-regulated gene following KSHV infection and a direct target of KSHV miRNAs. Conversely, Dicer and TRBP expression is increased following KSHV infection, which may promote increased amounts of the miRLC available for host and viral miRNAs. KSHV-mediated alterations in global miRNA biogenesis represent a novel mechanism by which KSHV evades an antagonizing host response.

## Results

### MCPIP1 Cleaves Human and Viral Pre-miRNAs

**Fig 1:**
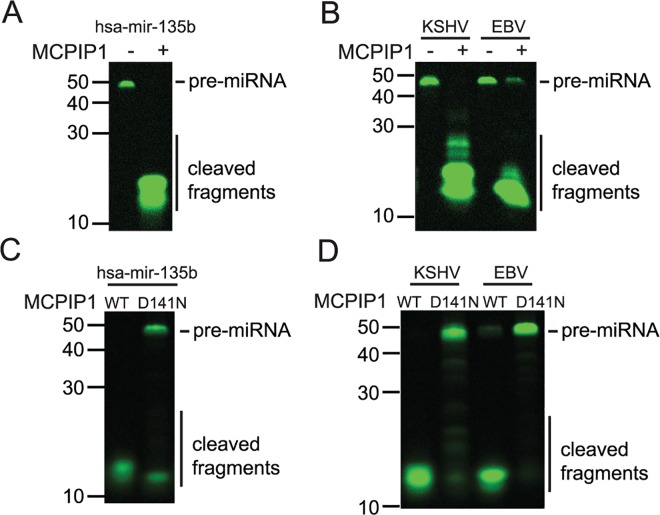
MCPIP1 directly cleaves human and viral pre-miRNAs. (A) In vitro cleavage assay for pre-hsa-mir-135b synthesized with 5’IRD800CWN labels and incubated with or without recombinant MCPIP1. Samples were resolved on a denaturing urea gel and scanned using an Odyssey scanner (LI-COR). Full-length pre-miRNAs and cleaved RNA products are indicated. (B) In vitro cleavage assay for pre-KSHV-mir-K12-3 and pre-EBV-mir-BART15 in the presence or absence of MCPIP1. (C) In vitro cleavage assay for pre-hsa-mir-135b with WT MCPIP1 or RNase dead, MCPIP1 D141N. (D) In vitro cleavage assays for pre-KSHV-mir-K12-3 and pre-EBV-mir-BART15 with WT or MCPIP1 D141N. Three independent in vitro cleavage assays were performed, and a representative image is shown for each condition.

MCPIP1 has previously been identified as an RNase and a suppressor of miRNA biogenesis that acts by cleaving the terminal loop of pre-miRNAs (pre-miRNAs) [16]. To determine whether MCPIP1 can directly cleave viral pre-miRNAs in addition to endogenous human pre-miRNAs, we developed an in vitro cleavage assay that utilizes fluorescently labeled synthetic pre-miRNAs (S1 Table) and recombinant MCPIP1 (S1 Fig). We found that MCPIP1 was able to efficiently cleave the fluorescently labeled precursor form of hsa-miR-135b (Fig 1A), as previously demonstrated [16]. We hypothesized that viral miRNAs, which are processed identically to human miRNAs, may also be degraded by MCPIP1. In vitro cleavage assays revealed that labeled KSHV-mir-K12-3 and EBV-mir-BART15 pre-miRNAs were efficiently cleaved in the presence of MCPIP1 (Fig 1B). We found that the mutant MCPIP1, D141N [15,16], which lacks RNase activity, significantly inhibited degradation of both human and viral pre-miRNAs indicating that the RNase activity of MCPIP1 is responsible for cleavage of the pre-miRNAs (Fig 1C and 1D).

**Fig 2:**
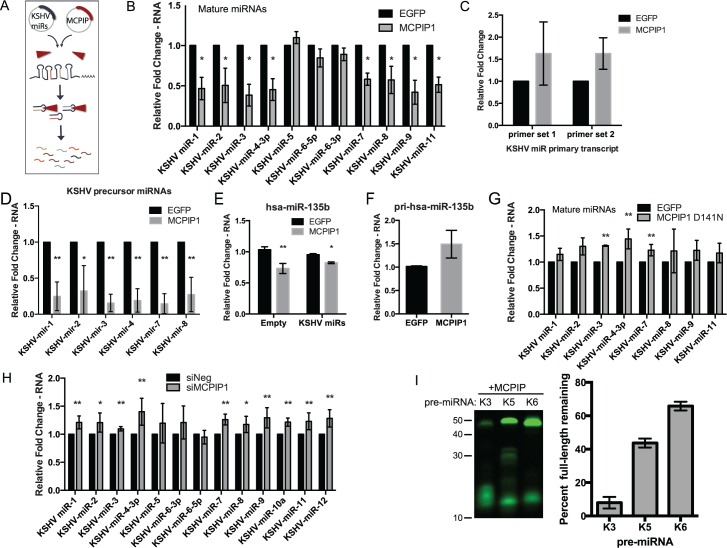
MCPIP1 represses mature miRNA expression by cleaving pre-miRNA. (A) Experimental design schematic for Fig 2B–2G. (B) 293 cells were transfected with a KSHV miRNA expression vector and a MCPIP1 expression plasmid. Expression of mature miRNA was measured using qPCR assays. Results are shown relative to RNU48 and normalized to the cells transfected with KSHV miRNA expression vector and the EGFP control. *N* = 3. (C) Expression of the KSHV miRNA primary transcript was measured from the same RNA samples as in (B) by qPCR using two different SYBR green primer sets. Results are shown relative to GAPDH, *N* = 3. (D) RNA from (B) was assayed for expression of KSHV pre-miRNAs using qPCR. Results are normalized to U6 and the EGFP control, *N* = 3. (E) Expression of hsa-miR-135b was measured from the same RNA samples as in (B) and normalized to the empty vector controls, *N* = 3. (F) Primary transcript level of hsa-miR-135b was measured using qPCR as in (B). Results are shown relative to β-actin and normalized to empty vector controls, *N* = 3. (G) 293 cells were transfected with a KSHV miRNA expression vector and an RNase dead, MCPIP1 D141N expression plasmid as in (B). *N* = 4. (H) BCBL-1 cells were transfected with siRNA to MCPIP1 followed by isolation of newly synthesized labeled RNA. cDNA was obtained and mature KSHV miRNA expression was measured by qPCR. Results are shown relative to RNU48 and normalized to the siNeg control. *N* = 3–5. (I) In vitro cleavage assay for pre-KSHV-mirs-3, 5, and 6, synthesized with 5’IRD800CWN labels. Pre-miRNAs were incubated with MCPIP1 and assayed for degradation. *N* = 3. The percent of full-length pre-miRNAs is shown in the accompanying graph. For all graphs, results are shown as mean ± SD (standard deviation). Significance was assessed using a Student’s *t* test, *p ≤ 0.05, **p ≤ 0.01. Numerical data can be found in S1 Data.

To determine if MCPIP1 can cleave KSHV pre-miRNAs within the context of a cellular environment, we transfected HEK293 cells with a KSHV miRNA expression vector and a MCPIP1 expression vector (Fig 2A). The miRNA expression was measured 72 hours post transfection. We observed a significant decrease in mature KSHV miRNA expression for a majority of the KSHV miRNAs measured when MCPIP1 is ectopically expressed as compared to the empty Enhanced Green Fluorescent Protein (EGFP) control (Fig 2B). Furthermore, expression of the KSHV miRNA primary transcript from which the majority of the KSHV miRNAs originate remains unchanged in the presence of MCPIP1 (Fig 2C). And the viral pre-miRNA expression decreased dramatically in the presence of MCPIP1 (Fig 2D). Together, these indicate that MCPIP1 is cleaving KSHV miRNAs at the pre-miRNA level and not affecting transcription of the clustered miRNA primary transcript. Expression of hsa-miR-135b is also significantly reduced in the presence of MCPIP1 while there is no decrease in pri-hsa-miR135b expression in concordance with Suzuki et al. (Fig 2E and 2F) [16]. A similar experiment with the MCPIP1 D141N mutant results in no decrease in mature KSHV miRNA expression levels, supporting our results from the in vitro cleavage assays (Figs 2G, 1D and S2A Fig).

In Fig 2, we observed the difference in MCPIP1-mediated degradation of pre-miRNAs to be greater than the changes in mature miRNA expression. This is likely due to differences in miRNA stability. Mature miRNAs have a half-life of approximately 5 d when incorporated in the RISC complex [17], while pre-miRNAs are thought to have a shorter half-life more similar to mRNA [18]. In order to test the effects of repression of MCPIP1 in KSHV-infected cells, we used siRNAs to reduce MCPIP1 expression in the KSHV-infected B-cell line, BCBL-1, and found no change in mature miRNA expression (S2 Fig). However, this assay is measuring expression of two classes of miRNAs: (1) miRNAs generated before and (2) miRNAs generated after MCPIP1 repression, and we next wanted to examine only newly transcribed miRNAs. In these assays, reduction of MCPIP1 using siRNA in BCBL-1 cells followed by purification of newly transcribed RNA transcripts showed increased expression of a majority of the mature KSHV miRNAs (Fig 2H). These data demonstrate not only that reduced MCPIP1 expression increases mature miRNA levels, but that this effect is primarily due to MCPIP1-mediated effects on pre-miRNAs during miRNA biogenesis as opposed to mature RISC-associated miRNA.

Finally, MCPIP1 did not cleave all KSHV pre-miRNAs with the same efficiency. Although MCPIP1 appeared to cleave most of the KSHV pre-miRNAs, it did not effectively cleave KSHV-pre-mir-5 or 6 (Fig 2I), which was consistent with the cellular assays (Fig 2B). Taken together, MCPIP1 in a purified system displays specificity for certain pre-miRNAs, but the determinants of specificity are currently unknown and require further investigation.

### KSHV Infection Affects Expression of the miRNA Biogenesis Machinery

**Fig 3:**
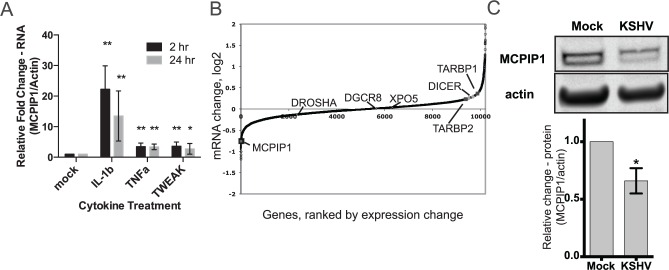
KSHV infection affects expression of miRNA biogenesis machinery. (A) HUVECs were treated with cytokines: IL-1β, TNFα, and TWEAK. Total RNA was harvested at 2 or 24 h after treatment, and MCPIP1 expression was measured using qPCR. Results are shown relative to β-actin and normalized to the untreated control. *N* = 5–11 per group. (B) Host mRNA expression data in KSHV latently infected HUVECs (48 hpi) [20]. Expression changes of Dicer, MCPIP1, TARBP1, and TARBP2 are indicated. (C) Western blots for MCPIP1 protein are shown for HUVECs that were either mock-infected or infected with KSHV, *N* = 3. For all graphs, results are shown as mean ± SD. Significance was assessed using a Student’s *t* test, *p ≤ 0.05, **p ≤ 0.01. Numerical data can be found in S1 Data.

MCPIP1 expression is induced by a number of proinflammatory cytokines, many of which are known to play a role in KS biology and are up-regulated after KSHV infection [12,13,19]. Accordingly, we found that IL-1β, TNFα, and tumor necrosis factor-like weak inducer of apoptosis (TWEAK)—a member of the TNF superfamily—can induce MCPIP1 mRNA expression in primary human umbilical vein endothelial cells (HUVECs) (Fig 3A). In the absence of inducing stimuli, MCPIP1 expression is maintained at lower levels. Our previously published microarray dataset [20] identified *MCPIP1* as a down-regulated gene following latent KSHV infection (Fig 3B and 3C). We note this repression is likely to be more significant at sites of infections in humans where inflammatory cytokines are present and driving increased expression of MCPIP1. In contrast to MCPIP1, essential miRNA biogenesis genes Dicer and TRBP (TARBP2) were up-regulated after latent KSHV infection (Fig 3B). We validated these observations following latent KSHV infection in HUVECs (S3 Fig). We also evaluated expression of these key biogenesis factors in MC116.219, a human B lymphocyte cell line (MC116 cells) that was infected with rKSHV.219 and maintained in the latent phase [21]. We found that MCPIP1 mRNA expression is reduced in infected MC116.219 cells, while Dicer and TRBP expression were both increased upon KSHV infection (S3 Fig). We found no significant changes in the expression of other key miRNA biogenesis proteins (Drosha, DGCR8, and Exportin 5) in the miRNA biogenesis pathway (Fig 3B). In conclusion, we observed alterations in expression of a number of miRNA biogenesis factors (MCPIP1, Dicer, TRBP) following KSHV infection in multiple relevant cell types.

### KSHV miRNAs Target the MCPIP1 3’ UTR

**Fig 4:**
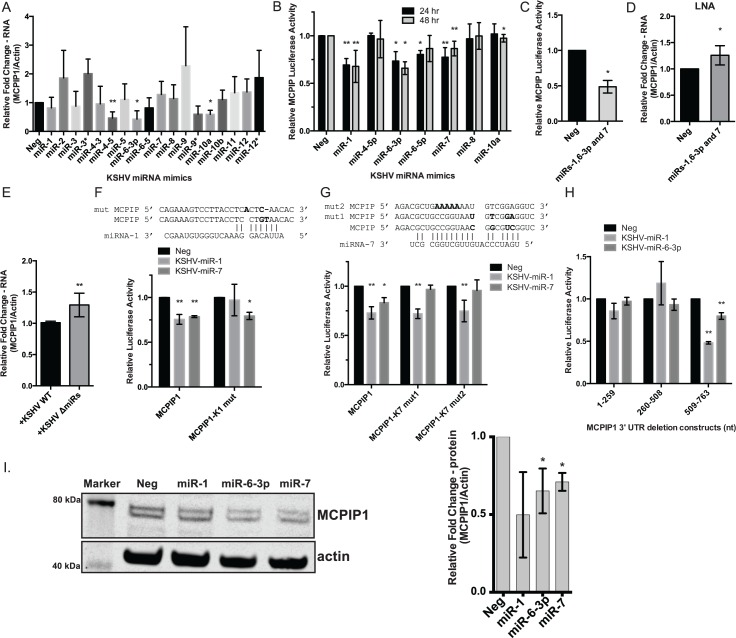
KSHV miRNAs target the MCPIP1 3’ UTR. (A) MCPIP1 RNA expression was determined using qPCR from HUVECs transfected with individual KSHV miRNA mimics. Results are shown relative to β-actin and normalized to the negative control, *N* = 2–7. (B) 293 cells were transfected with individual KSHV miRNAs and a MCPIP1 3’ UTR reporter plasmid. Lysates were analyzed 24 or 48 hpt, and relative MCPIP1 luciferase activity is expressed as a ratio to an internal luciferase control and normalized to an empty reporter vector and the negative control miRNA, *N* = 3–9. (C) 293 cells were transfected with a combination of KSHV miRNA mimics, KSHV-miR-1, 6-3p and 7 as in B, *N* = 3. (D) BCBL-1 cells were nucleofected with LNAs for KSHV-miR-1, 6-3p and 7. RNA was isolated 24 hpt and MCPIP1 expression was measured using qPCR. *N* = 4. (E) MCPIP1 expression was measured using qPCR as in (A) from iSLK cells containing a WT KSHV genome and a mutant containing a KSHV miRNA cluster deletion. Results are shown relative to β-actin and normalized to the WT KSHV infected iSLK cells, *N* = 6. (F) The sequence of the KSHV-miR-1 binding site (mutations in bold) in the MCPIP1 3’UTR and luciferase reporter assays containing a mutated binding site for KSHV miR-1. *N* = 3–4. (G) The sequence of the KSHV-miR-7 binding site (mutations in bold) in the MCPIP1 3’UTR and luciferase reporter assays containing two different mutations for the KSHV miR-7 binding site, *N* = 4–9. (H) 293 cells were transfected with individual KSHV miRNAs and one of the three MCPIP1 deletion reporter vectors. *N* = 3. (I) Western blots are shown for HUVECs transfected with a control (Neg) or KSHV miRNA mimics, *N* = 3. For all graphs, results are shown as mean ± SD of at least three independent biological replicates. Significance was assessed using a Student’s *t* test, *p ≤ 0.05, **p ≤ 0.01. Numerical data can be found in S1 Data.

Since a subset of mature viral miRNAs may enter the cells via virions or exosomes [22,23], we hypothesized that one mechanism for reduced MCPIP1 expression immediately following KSHV infection may be through KSHV miRNAs targeting MCPIP1 mRNA directly. To determine the effect of KSHV miRNAs on MCPIP1 mRNA expression, individual miRNAs were transfected into HUVECs and MCPIP1 mRNA expression was determined by qPCR. Three KSHV miRNAs (KSHV-miR-4-5p, 6-3p, and 10a) resulted in decreased MCPIP1 mRNA expression (Fig 4A). Since miRNAs can silence genes through both mRNA degradation and translational repression [24], we also cloned the MCPIP1 3’UTR downstream of a luciferase reporter vector and transfected individual KSHV miRNAs into 293 cells along with the MCPIP1 luciferase reporter vector. KSHV miRs-1, 6-3p, and 7 demonstrated a statistically significant repression of a luciferase reporter containing the MCPIP1 3’ UTR (Fig 4B). Unrelated miRNAs tested as controls showed no change in reporter activity. Furthermore, when we transfected the combination of KSHV-miRs-1, 6-3p, and 7 with the MCPIP1 3’ UTR reporter vector, we observed the strongest decrease in MCPIP1 reporter activity (Fig 4C). To investigate miRNA-mediated MCPIP1 repression in the context of KSHV infection, we inhibited KSHV-miRNAs-1, 6-3p, and 7 using locked nucleic acid inhibitors (LNAs) in BCBL-1 cells. We observed an increase in MCPIP1 expression following repression of this combination of KSHV miRNAs (Fig 4D). Since multiple KSHV miRNAs are involved in the regulation of MCPIP1, we obtained iSLK mutant cells deleted for the KSHV miRNA cluster [25]. We found that expression of MCPIP1 was increased in the iSLK miRNA cluster mutant as compared to the wild type KSHV infected iSLK cells (Fig 4E), highlighting the importance of KSHV miRNA expression in the down-regulation of MCPIP1 expression.

The miRNA target prediction program, miRanda [26], identified a 7-mer m8 type KSHV-miR-1 potential target binding site in the MCPIP1 3’ UTR (Fig 4F and S2 Table). Following the mutation of this predicted binding site, KSHV-miR-1 no longer repressed MCPIP1 3’ UTR reporter expression, while reporter plasmids with the KSHV-miR-1 mutation could still be repressed by KSHV-miR-7 (Fig 4F). Our results are supported by previous PAR-CLIP experiments that identified MCPIP1 (ZC3H12A) as a KSHV-miR-1 target in KSHV-infected BC-1 and BC-3 cells [5]. Thus, not only is MCPIP1 a direct target of KSHV-miR-1 but this miRNA binding site is important for KSHV-miR-1 mediated repression of MCPIP1.

Although the miRanda program [26] was unable to find any additional miRNA binding sites in the MCPIP1 3’ UTR, we found a potential KSHV-miR-7 target site using an alternative miRNA target prediction program. RNA hybrid [27] identified a noncanonical KSHV-miR-7 potential target binding site with extensive 3’ pairing (Fig 4G). We mutated either the 5’ miRNA region (mut1) or the 3’ target site (mut2) and observed that KSHV-miR-7 could no longer repress either of these reporter constructs indicating that both of these interactions are critical for KSHV-miR-7 directed MCPIP1 repression (Fig 4G). While we could identify a few potential KSHV-miR-6-3p binding sites within the MCPIP1 3’ UTR, we observed little repression in the presence of KSHV-miR-6-3p. Therefore, we separated the MCPIP1 3’ UTR sequence into three segments (S3 Table). We determined that the KSHV-miR-6-3p binding site is within nucleotides 509–763 of the MCPIP1 3’ UTR along with the KSHV-miR-1 site (Fig 4H). We additionally analyzed MCPIP1 protein expression after transfection with KSHV miRNA mimics and found a correlation between repression of the luciferase reporters and repression of endogenous MCPIP1 protein (Fig 4I). Together, we were able to validate MCPIP1 as an mRNA target for KSHV miRNAs and demonstrate that these viral miRNAs can reduce MCPIP1 expression.

### Increased Expression of miRNA Biogenesis Components Dicer and TRBP after KSHV Infection

**Fig 5:**
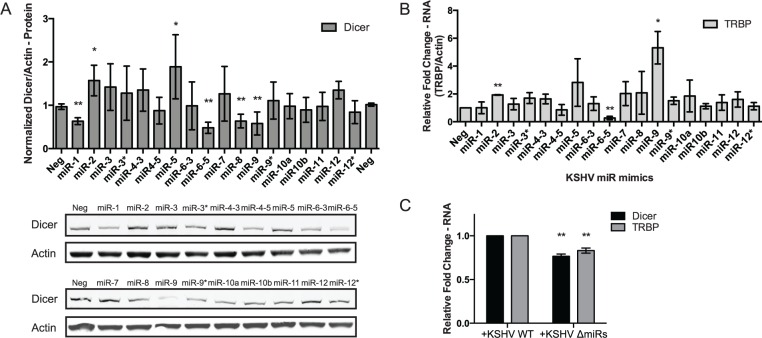
KSHV miRNAs increase expression of miRNA biogenesis components Dicer and TRBP. (A) Dicer protein expression was determined by western blot from HUVECs transfected with individual KSHV miRNA mimics. The results are averaged from 4–5 replicates and a representative western blot is shown. (B) TARBP2 RNA expression was determined using qPCR from HUVECs transfected with individual KSHV miRNA mimics. Results are shown relative to β-actin and normalized to the negative control. For all graphs, results are shown as mean ± SD, *N* = 3. Significance was assessed using a Student’s *t* test, *p ≤ 0.05, **p ≤ 0.01. (C) Dicer and TRBP expression was measured using qPCR as in (B) from iSLK cells containing a WT KSHV genome and a mutant containing a KSHV miRNA cluster deletion. *N* = 5. For all graphs, results are shown as mean ± SD. Significance was assessed using a Student’s *t* test, *p ≤ 0.05, **p ≤ 0.01. Numerical data can be found in S1 Data.

As opposed to MCPIP1, we also demonstrated an increase in Dicer expression following de novo KSHV infection (Fig 3 and S3 Fig). Additionally, previously published data showed an increase in Dicer mRNA levels following transfection with a combination of 16 KSHV miRNA mimics [28]. This suggests that the up-regulation of Dicer expression is partially due to the expression of KSHV miRNAs. Therefore, we transfected individual KSHV miRNAs into HUVECs and determined the expression of Dicer at both the mRNA and protein level. Focusing on miRNAs that increased Dicer expression (in accordance with our de novo infection data), we found that KSHV-miR-2 and 5 resulted in up-regulation in Dicer levels (Fig 5A and S4 Fig). Further, KSHV-miR-2 and 5 were also able to increase expression of the miRLC component and Dicer binding partner, TARBP2 (Fig 5B). Deletion of the KSHV miRNA cluster in the iSLK ΔmiRNA mutant cell line [25] resulted in decreased expression of Dicer and TRBP as compared to the wild type KSHV infected iSLK cells (Fig 5C). Taken together, these data indicate that KSHV miRNAs are involved in the regulation of Dicer and TRBP expression. The increase in expression of Dicer and TRBP is likely through repression of an inhibitor of these genes, since miRNAs are generally found to repress gene expression [29].

AU-binding factor-1 (AUF1), also known as heterogeneous nuclear ribonucleoprotein D (hnRNP D) lowers Dicer mRNA stability resulting in a reduction of Dicer abundance and mature miRNA expression levels [30]. We have observed decreased expression of AUF1 following de novo KSHV infection (S4 Fig). This suggests that latent viral gene products may stimulate Dicer expression by repressing AUF1 and likely additional repressors.

**Fig 6:**
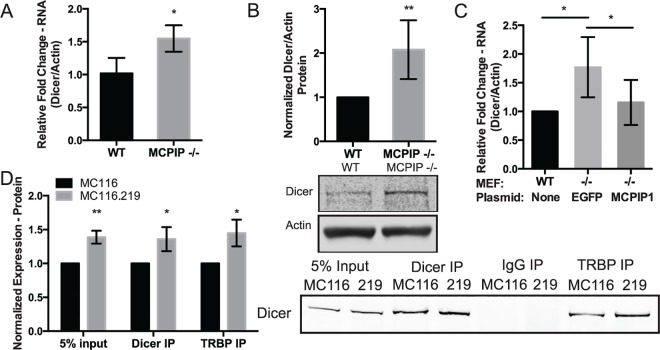
Repression of MCPIP1 increases Dicer expression and TRBP association. (A) RNA from WT and MCPIP1 -/- MEFs were analyzed for Dicer expression using qPCR. Results are shown relative to β-actin and normalized to the WT control. *N* = 3. (B) WT and MCPIP1 -/- MEF protein lysates were evaluated for Dicer using western blot analysis. β-actin was used as a loading control. The results are averaged for six replicates, and a representative western blot is shown. (C) WT and MCPIP1 -/- MEFs were transfected with a MCPIP1 expression vector or an EGFP negative control vector, and RNA was isolated 48 hpt. RNA was DNase treated and analyzed for Dicer expression using qPCR. Results are shown relative to β-actin and normalized to the WT control, *N* = 4. (D) MC116 and KSHV infected MC116.219 cells were immunoprecipitated with anti-Dicer and anti-TRBP antibodies and blotted for Dicer. The results are averaged for four replicates and a representative blot is shown. For all graphs, results are shown as mean ± SD. Significance was assessed using a Student’s *t* test, *p ≤ 0.05, **p ≤ 0.01. Numerical data can be found in S1 Data.

In addition to KSHV-miRs-2 and 5 that up-regulate Dicer expression, we have also observed increased Dicer expression following the down-regulation of MCPIP1 (S4 Fig). Conversely, the knockdown of Dicer has no effect on MCPIP1 expression (S4 Fig). MCPIP1 knockout mouse embryonic fibroblasts (MEFs) also express more Dicer at both the RNA and protein level as compared to WT MEFs (Fig 6A and 6B). Addition of a MCPIP1 expression vector back into the MCPIP1 -/- MEFs (S4 Fig) was able to rescue the phenotype and restore Dicer expression to wild type levels (Fig 6C). These data reveal that expression of KSHV miRNA-2 and 5, or separately, repression of MCPIP1 can increase Dicer expression. This suggests two different and distinct mechanisms for the observed increase in Dicer expression following KSHV infection.

Expression of essential miRNA biogenesis players Dicer and TRBP are increased following latent KSHV infection, suggesting an increase in the core components of the miRNA loading complex (miRLC) that is available for miRNA processing. Coimmunoprecipitations of Dicer and TRBP in uninfected MC116 cells and KSHV infected MC116.219 cells demonstrated increased precipitation of Dicer with TRBP in infected cells compared to uninfected cells (Fig 6D). This may suggest an increase in miRLC formation that could allow increased loading of mature miRNAs into RISC as they move through the biogenesis pathway due to increased amounts of the intermediate miRLC available as well as an increased stability of miRNAs as they are incorporated into the final RISC complex [31,32]. However, the rate-limiting factor in miRLC formation remains unknown.

### MCPIP1 Is Repressed by KSHV Infection in the Presence of Stimulating Cytokine, IL-1β

**Fig 7:**
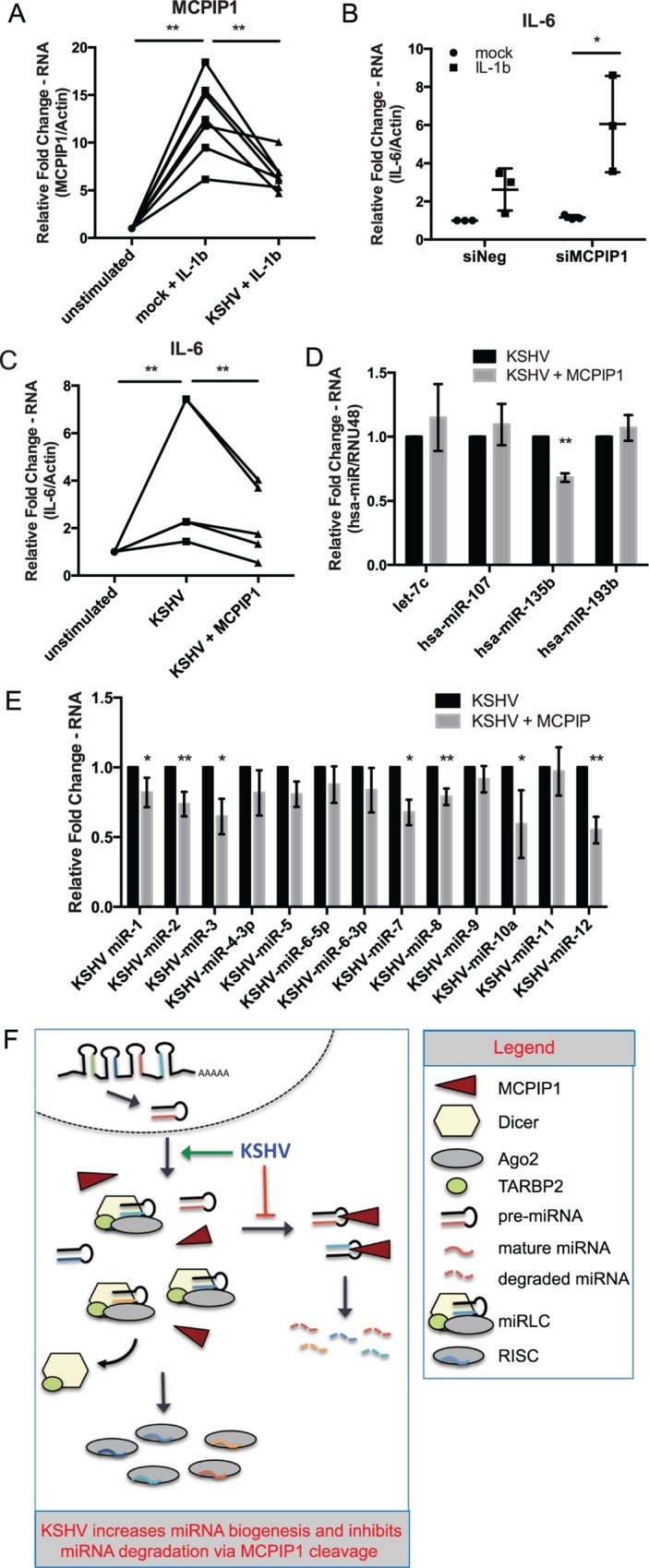
MCPIP1 degrades target mRNA and viral miRNAs following KSHV infection. (A) Mock- and KSHV-infected HUVECs (7 dpi) were stimulated with IL-1β for 24 h, and RNA was isolated. MCPIP1 was measured using qPCR. Results are shown relative to β-actin and normalized to the mock infected, untreated control. The unstimulated control was set to a default value of 1 for each replicate. Significance was assessed using a paired Student’s *t* test, *N* = 7. (B) BCBL-1 cells were nucleofected with siRNA to MCPIP1 followed by stimulation with IL-1β for 24 h. RNA was isolated, and IL-6 mRNA expression was measured using qPCR. Results are shown relative to β-actin and normalized to the siNeg, untreated control, *N* = 3. (C) 293 GFP-MCPIP1 cells were induced to express MCPIP1 followed by KSHV infection and RNA was isolated 3 dpi. IL-6 mRNA expression was measured using qPCR. Results are shown relative to β-actin and normalized to the mock infected, untreated control. The unstimulated control was set to a default value of 1 for each replicate. Significance was assessed using a paired Student’s *t* test, *N* = 5. (D) GFP-MCPIP1 was induced followed by KSHV infection as in (C), and mature human miRNA expression was assessed using qPCR assays. Results are shown relative to RNU48 and normalized to the KSHV infected samples, *N* = 5. (E) GFP-MCPIP1 was induced followed by KSHV infection as in (C), and mature KSHV miRNA expression was assessed using qPCR assays. Results are shown relative to RNU48 and normalized to the KSHV infected samples, *N* = 2–5. All results are shown as mean ± SD. Significance was assessed using an unpaired Student’s *t* test, *p ≤ 0.05, **p ≤ 0.01. (F) Schematic representation of the model showing that KSHV infection increases miRNA biogenesis through increased expression of miRLC core components and inhibition of MCPIP1-mediated miRNA degradation. Numerical data can be found in S1 Data.

We have demonstrated that a number of cytokines including IL-1β can induce MCPIP1 mRNA expression (Fig 3A) and upon latent KSHV infection in primary endothelial cells, we report that MCPIP1 is decreased (Fig 3B). To test whether KSHV infection could counteract cytokine-induced MCPIP1 induction, we infected HUVECs with KSHV for 7 d and then treated cells with IL-1β for 24 h. We found that MCPIP1 was induced at a significantly lower level in the KSHV-infected cells compared to mock-infected cells (Fig 7A). We hypothesize that latently expressed KSHV miRNAs may be partially responsible for the reduced expression of MCPIP1, but latent proteins may also contribute. These findings support our hypothesis that MCPIP1 is repressed by KSHV infection in both the absence and the presence of stimulating cytokines.

### MCPIP1 Degrades Target mRNA and Viral miRNAs following KSHV Infection

Infection with KSHV up-regulates IL-6 expression, a known autocrine growth factor for KS cells [33]. In contrast, MCPIP1 destabilizes IL-6 through its RNase activity, targeting a stem loop structure within the IL-6 3’ UTR [15,34]. To determine the consequences of MCPIP1 loss of function in the context of KSHV infection, MCPIP1 was repressed with siRNAs in KSHV positive BCBL-1 cells (S5 Fig) followed by IL-1β treatment for 24 h. We found that induction of IL-6 by IL-1β was stronger when MCPIP1 was repressed (Fig 7B). These data demonstrate that reduced MCPIP1 expression can relieve the MCPIP1-mediated reduction of its mRNA target, IL-6, in the presence of a stimulating cytokine, IL-1β. Conversely, we determined the effect of induced MCPIP1 expression in the context of de novo KSHV infection. A doxycycline-inducible transgene harboring the coding sequence of MCPIP1 fused to GFP was introduced in HEK293TRex cells. GFP-MCPIP1 was induced (S5 Fig) followed by KSHV infection. Overexpression of MCPIP1 in the presence of KSHV infection results in significantly decreased levels of IL-6 (Fig 7C).

We also measured expression of both human and viral mature miRNAs in MCPIP1-induced 293 cells infected with KSHV. While the expression of multiple human miRNAs remained unchanged after MCPIP1 induction, expression of mature hsa-miR-135b was significantly reduced (Fig 7D). In contrast to many of the human miRNAs, we found that increased MCPIP1 expression resulted in significantly lower levels of many KSHV miRNAs (Fig 7E). We also observed increased LANA expression and a decrease in some lytic genes, ORF69 and K14 (S5C Fig), suggesting that MCPIP1 expression can have consequences to viral protein-encoding genes as well. Together, this data shows that modulation of MCPIP1 in the context of KSHV infection can result in significant changes in expression for its mRNA and miRNA targets and supports a role for MCPIP1 in KSHV pathogenesis.

## Discussion

While MCPIP1 cleaves both human and viral pre-miRNAs, there appears to be some level of specificity that remains unclear (Figs 2B, 7D and 7E). We hypothesize that this could include a number of different possibilities including: (1) MCPIP1 cleaves pre-miRNA and not mature miRNAs, (2) some pre-miRNAs are sensitive to MCPIP1 while others are not, and (3) steady state expression of host miRNA (as mature miRNA incorporated into RISC has a half-life of 5 d [17]) as compared to newly synthesized viral miRNA. At the time of infection, most human miRNAs exist in the mature miRNA form in the RISC complex. However, incoming viral miRNAs are distinct, because these viral miRNAs start at the beginning of the biogenesis pathway, which makes them more sensitive to MCPIP1 since it cleaves pre-miRNAs, but not mature miRNAs. Recent work by Mino et al. showed that MCPIP1 recognizes and degrades transcriptionally active mRNA targets [35]. Together with our data, this suggests that reduced MCPIP1 expression increases mature miRNA levels (Fig 2H) and is primarily due to MCPIP1-mediated effects during miRNA biogenesis as opposed to mature RISC-associated miRNA. An additional consideration to explain differences in MCPIP1-mediated cleavage efficiency of pre-miRNAs includes differences in nuclear transport. However, the correlation between the amount of MCPIP1-mediated degradation of specific pre-miRNAs in our cell-free assays (without transport considerations) in Fig 1 and cellular assays in Fig 2 suggests that this is not the case, at least for the viral miRNAs that we investigated. Additionally, we observed MCPIP1-mediated repression of miRNAs from eight of the ten KSHV pre-miRNAs, with some of these pre-miRNAs from all three levels of expression (high, moderate, and low) in the O'Hara et al. pre-miRNA expression data [18]. The two pre-miRNAs that were resistant to MCPIP1 degradation, -K5 and -K6, were from two classes of expression (moderate and low). Taken together, these data suggest that there is not a strong correlation between pre-miRNA expression and sensitivity to MCPIP1 degradation. Further investigation of MCPIP1 cleavage targets and specificity will yield interesting insights into MCPIP1 specificity.

In addition to pre-miRNAs, MCPIP1 is reported to regulate expression of many proinflammatory cytokines, including IL-6 (Fig 7) [15]. Recently, Mino et al. showed that MCPIP1 recognizes and degrades transcriptionally active mRNA targets through stem loop structures within the 3’ UTR [35]. Although increased IL-6 expression may initially seem to benefit only the host immune system, multiple studies have shown that IL-6 has beneficial properties to KSHV infection. Human IL-6 can promote growth of KSHV-infected PEL cell lines [36] and AIDS-KS-derived cells [33]. In addition, KSHV has evolved to express its own IL-6 homolog, viral IL-6 (ORF-K2) [37], and a latently expressed protein, LANA, can promote expression of IL-6 (PMID: 11781250). Elevated human IL-6 levels also correlate with increased KSHV viral load and worse clinical outcome [38]. These results suggest that KSHV-mediated control of MCPIP1 and IL-6 expression can benefit KSHV infection.

In this study, we report that KSHV infection inhibits MCPIP1, a negative regulator of miRNA biogenesis, and up-regulates critical miRNA processing components Dicer and TRBP to evade host mechanisms that inhibit expression of viral miRNAs. We suggest that this is a complex interaction between the host immune defense and the virus. MCPIP1 is a potent regulator of the innate immune response and possesses antiviral activities [15,39–41]. MCPIP1 was previously found to target multiple viral RNA species including hepatitis C virus (HCV), Japanese encephalitis virus (JEV), dengue virus (DEN) and HIV resulting in decreased steady states levels of the viral RNA due to its RNase activity [40–42]. Conversely, the virus wants to repress any host defenses that down-regulate viral RNAs to allow for viral replication, viral dissemination, or persistent viral infection. Multiple pieces of evidence suggest that MCPIP1 may inhibit miRNA biogenesis during other viral infections as well. EBV is a γ-herpesvirus that is related to KSHV and expresses its own viral miRNAs. Similar to our findings, a recent paper found that EBV-infected proliferating B cells exhibit a significant repression of MCPIP1 compared to EBV-negative purified adult human CD19+ peripheral blood B cells [43]. In addition to the KSHV miRNAs reported here that target the MCPIP1 3’ UTR, miRanda [26] predicts four EBV miRNAs including EBV-miR-BART8-5p, BART8-3p, BART10-5p, and BART11-5p that may target the MCPIP1 3’UTR resulting in repression. In this report, we focused on changes to miRNA biogenesis factors mediated by viral miRNAs, but it is also possible that latent viral proteins and virally induced changes in host mRNAs and miRNAs could also help establish a favorable environment for viral miRNA biogenesis. Further studies are required to elucidate if simultaneous repression of MCPIP1 and induction of miRNA pathway components is a common strategy of viruses that encode miRNAs to ensure biogenesis of their viral miRNAs and counteract the host defense.

One possible model for the KSHV-mediated changes observed in the miRNA biogenesis pathway is represented in Fig 7E. It is likely that both the repression of inhibitory miRNA biogenesis factors and the induction of productive biogenesis factors are important for KSHV infection and the production and expression of viral miRNAs. We propose that KSHV inhibits MCPIP1 and up-regulates Dicer to evade host mechanisms of inhibiting expression of foreign miRNAs. We inspected PAR-CLIP data for cellular genes that may reveal mechanisms of miRNA-mediated changes in Dicer1 expression. We first searched the literature for reported repressors of Dicer1 (CALCOCO2, PIWIL1, DDB1, and CASP3). We searched PAR-CLIP hits for these genes and did not find any KSHV miRNA hits for PIWIL1 or DDB1. We did find KSHV miRNA hits for CALCOCO2 (miR-K10A) and CASP3 (miR-K1, -K3, -K4-3p), but not for miR-K2 or miR-K5. This current report aims to provide insight into the comprehensive understanding of miRNA processing in the context of KSHV infection. KSHV-mediated alterations in miRNA biogenesis factors represent a novel mechanism by which KSHV interacts with its host, and a new mechanism for the regulation of miRNA expression. Our results suggest that this may be part of a general viral response to overcome host defense mechanisms.

## Materials and Methods

### Cell Culture and Reagents

HEK293 cells and A549 cells were obtained from the ATCC and maintained in Dulbecco’s Modified Eagle’s medium (DMEM) supplemented with 10% FBS (Sigma) and a penicillin and streptomycin and glutamine solution (Life Technologies). HUVECs (Lonza) were maintained in EGM-2 media (Lonza) for up to five passages. MC116 and MC116.219 cells are uninfected and KSHV-infected cell lines, respectively [21], and cultured with RPMI 1640 media (Life Technologies) supplemented with 20% FBS and a penicillin, streptomycin and glutamine solution (Life Technologies). MC116.219 cells were maintained in 10 ug/ml puromycin. MCPIP -/- and WT MEFs were previously described [44,45] and cultured in DMEM medium with 10% FBS and a penicillin and streptomycin and glutamine solution (Life Technologies). Dox-inducible GFP-MCPIP1 HEK293 cells were cultured in DMEM with 10% Tetracycline-free FBS and a penicillin and streptomycin and glutamine solution (Life Technologies) and maintained with 1 ug/ml puromycin and 200 ug/ml G418 as described previously [41]. These cells were induced with Doxycycline (10 ng/ml) to express GFP-MCPIP. iSLK and iSLK mutant cells deleted for the KSHV miRNA cluster were obtained from the Renne lab [25]. iSLK cells are cultured in DMEM medium with 10% FBS and a penicillin, streptomycin and glutamine solution (Life Technologies) with 1 ug/ml puromycin and 250 ug/ml G418. iSLK mutant cells were also maintained in 1200 ug/ml hygromycin. All cultures were grown at 37°C with 5% CO_2_. Synthetic KSHV miRNA mimics were obtained from Ambion. ON-TARGETplus SMARTpool small interfering RNAs (siRNAs) targeting DICER and MCPIP1 and an ON-TARGETplus nontargeting pool were obtained from Thermo Fisher Scientific. The cytokines IL-1β, TNFα and TWEAK were purchased from Peprotech and used at a working concentration as follows: IL-1β (15 ng/ul), TNFα (15 ng/ul) and TWEAK (500 ng/ul). Power-LNAs against KSHV miRNAs- miR-K12-1, -6-3p and–K7 were obtained from Exiqon.

### Microarray Data

Microarray data can be found at http://dx.doi.org/10.6084/m9.figshare.1446120.

### Plasmids

MCPIP1 was expressed using the pReceiver-M11-MCPIP1 (GeneCopeia). Primers to generate MCPIP1 D141N are as described in S3 Table. The KSHV miRNA expression plasmid was generated as previously described [46], and primers are listed in S3 Table. The 3’UTR of MCPIP1 (ZC3H12A) used for luciferase reporter assays was obtained from SwitchGear Genomics (product ID: S806820) and expressed in pLightSwitch_3UTR. The MCPIP1 3’UTR was amplified using the pLS primers outlined in S3 Table and cloned into the vector pCR8/GW/TOPO using a TA cloning kit (Invitrogen). Positive clones were verified and a LR clonase II (Invitrogen) recombination reaction catalyzed the transfer into the destination vector, pDEST765. pDest765 expresses firefly luciferase driven by the herpes simplex virus thymidine kinase (TK) promoter as an internal control and *Renilla* luciferase driven by a simian virus 40 (SV40) promoter. This 3’UTR reporter vector is designated pDest765-MCPIP1. To identify KSHV miRNA target sites within the MCPIP1 3’UTR, site-directed mutagenesis was performed on the MCPIP1 3’ UTR plasmid using the QuikChange II Site-Directed Mutagenesis kit (Agilent Technologies). Primers to generate the MCPIP1 site-directed mutants are described in S3 Table. Deletion constructs of MCPIP1 3’ UTR were constructed using gBlocks gene fragments as described in S4 Table (IDT Technologies). Briefly, gBlocks were subjected to a tailing reaction with dATP to add a 3’ A followed by TA cloning into pCR8/GW/TOPO (Invitrogen). Positive clones were verified and a LR clonase II (Invitrogen) recombination reaction catalyzed the transfer into the destination vector, pDest765. Three deletion constructs (~260 nt each) were made that span the ~800 nt MCPIP1 3’UTR.

### RNA Isolation and cDNA Synthesis

mRNA and miRNA was isolated together using miRNeasy mini kit (Qiagen). cDNA for mRNA quantification was prepared from 1 ug of RNA using the High–capacity cDNA reverse transcription kit (Applied Biosystems) with random primers. mRNA cDNA was then diluted 1:5 in water and 4 ul was used for real time PCR. For quantitation of KSHV-pre-miRNAs, RNA was isolated using the miRNeasy mini kit (Qiagen). 1 ug of RNA was then DNase treated using the TURBO DNA-free kit (Life technologies) and used for cDNA synthesis via the High–capacity cDNA reverse transcription kit (Applied Biosystems) with random primers. cDNA was diluted 1:5 before real time PCR. For quantitation of mature miRNAs (human and viral), 30–40 ng of RNA was reverse transcribed with specific RT primers using the MicroRNA Reverse Transcription Kit (Applied Biosystems). The primer-specific cDNA for miRNAs was then diluted 1:6.66 in water.

### RNA Capture and cDNA Synthesis

The Click-iT nascent RNA capture kit (Molecular probes by Life Technologies) was utilized to selectively separate newly synthesized RNA from existing RNA. To this end, BCBL-1 cells were nucleofected (see above protocol) with siRNA to MCPIP1 or a siNeg control. Approximately 24 h after nucleofection with siRNA, 5-ethynyl Uridine (EU) from the RNA capture kit was added to the BCBL-1 cultures. EU remaining in culture with the cells overnight (~18 h), and RNA was harvested using the miRNeasy mini kit (Qiagen). These samples will contain both unlabeled and EU-labeled RNA. 1–2 ug of RNA was then used for a copper catalyzed reaction that creates a biotin-based handle to capture EU-labeled RNA. The RNA was then precipitated, and concentration was measured using a NanoDrop 1000 (ThermoFisher). 250 ng of biotinylated RNA was used for each streptavidin pull-down of biotinylated RNA. cDNA was then immediately prepared from the RNA pull down using the High–capacity cDNA reverse transcription kit (Applied Biosystems) with random primers for mRNA. cDNA from the KSHV miRNAs was reverse transcribed with specific RT primers using the MicroRNA Reverse Transcription Kit (Applied Biosystems). Primers for five small RNAs were used per RNA pull down reaction. cDNA was then diluted 1:2 in water and 4 ul was used for real time PCR as described below. Average and standard deviations are calculated for at least three biological replicates.

### Real-Time PCR

Real Time qPCR was performed on an ABI StepOnePlus real time PCR system (Applied Biosystems). All qPCR reactions contained 3–5 ul of diluted cDNA. qPCR cycling conditions started with a 2-min activation step at 50°C, 10 min template denaturation step at 95°C, followed by 40 cycles of 95°C for 15 s and 60°C for 1 min. SYBR green assays also included a melt curve at the end of the cycling protocol with continuous fluorescence measurement from 60°C to 95°C.

The mature KSHV miRNA were quantified using 5 μl of diluted cDNA and microRNA primer probe sets (S4 Table, Applied Biosystems) with Universal PCR Master Mix, no AmpErase UNG (Applied Biosystems) as described by the manufacturer. Real-time PCR for human mRNA and miRNA was performed using 5 μl of diluted cDNA with primer probe mixtures (S4 Table) and Universal PCR Master Mix, no AmpErase UNG (Applied Biosystems) as described by the manufacturer. KSHV markers were quantified with real time qPCR using SYBR green primers as described in S4 Table at a final concentration of 300 nM. All SYBR green real time PCR reactions contained 3 ul of diluted cDNA in a 25 ul total reaction volume with FastStart universal SYBR green master mix (Roche). Standard qPCR cycling conditions were used as above. Relative gene expression was determined using the comparative C_T_ method and normalized to β-actin. Data is normalized to the untreated control and reported as fold change. Results are presented as the gene of interest expression levels normalized to control levels, relative to negative controls. Average and standard deviations are calculated for at least three biological replicates.

### Recombinant MCPIP1

We used recombinant protein from two sources. One source was an in vitro wheat germ expression system (Abnova). The other source used an *Escherichia coli* expression culture system (Leidos). Both systems used a GST tag and were purified using glutathione beads.

### In Vitro Cleavage Assays

Pre-miRNA sequences were synthesized with 5’IRD800CWN labels. The sequences of the pre-miRNAs can be found in S1 Table. Labeled pre-miRNAs (20 nmoles) were incubated with either in vitro translated MCPIP1 (20 ng, Abnova) or *E*. *coli* expressed and purified protein (200 ng) in 15 μl reactions in 5 mM MgCl_2_, 0.5 mM ATP, 100 mM creatine phosphate, 20 mM Tris pH 7.5, 100 mM KCl, 10% glycerol, 5 mM DTT. Reactions were incubated at 37° for 2 h and loaded onto a 15% denaturing urea gel. A DNA ladder was stained with SYTO 60 (Life Technologies) for size markers. Gels were scanned using an Odyssey scanner (LI-COR). Gels were quantified using LI-COR Image Studio software. Percent of full-length pre-miRNAs was calculated as the total signal per lane minus the amount of degraded RNA and multiplied by 100.

### KSHV Production and Latent KSHV Infection

KSHV virus supernatant was prepared from BCBL-1 cells as described previously [47]. HUVECs were infected with KSHV in media containing 8 ug/ml Polybrene for 6 h at 37°C. RNA or whole-cell lysates were prepared at 7 d post infection (dpi) for analysis by real time PCR or western blotting as previously described [47]. Doxycycline- inducible GFP-MCPIP1 HEK293 cells were induced with Dox for 24 h to express GFP-MCPIP1 followed by KSHV infection (KSHV prep from BCBL-1 cells). Doxycycline was replaced after infection and mRNA and miRNA expression was measured 72 hpi (h post infection).

### Luciferase Reporter Assays

3′UTR luciferase assays were performed as previously described [48]. Briefly, 3′UTRs were cloned downstream of the renilla luciferase gene reporter. KSHV miRNA mimics were reverse transfected at a concentration of 1–3 pmol per well along with luciferase reporter vectors into HEK293 cells with Lipofectamine 2000 (Invitrogen). Individual KSHV miRNA mimics were transfected at 2–3 pmol/well while combinations of KSHV miRNA mimics were transfected at 1 pmol/well for a total concentration of 3 pmol/well. Reporter assays were performed using the Dual-Luciferase reporter system (Promega) at 24 and 48 hpt. Luciferase values were normalized to an internal SV40-driven firefly luciferase gene and to parental vectors lacking cloned 3′UTRs. Relative MCPIP1 luciferase activity was calculated by determining the ratio of *Renilla*/firefly luciferase followed by normalization to both the empty luciferase vector control and the negative control miRNA mimic. Each transfection was performed at least three independent times and was assayed in triplicate. Results are presented as the mean ± SD of at least three biological replicates.

### Western Blot Analysis

Total cell protein was harvested using RIPA lysis buffer (Sigma) supplemented with 1X Halt protease and phosphatase inhibitor cocktail (Thermo Scientific). Protein concentration was determined using Quick Start Bradford Dye Reagent 1X (Bio-Rad), and equal amounts of protein were loaded onto a NuPAGE 4%–12% Bis-Tris Gel (Invitrogen) followed by transfer onto a nitrocellulose membrane (Invitrogen). The LI-COR Odyssey system was used for the detection and quantitation of protein bands. Primary antibodies include: mouse anti-Dicer (ab14601, Abcam), rabbit anti-TRBP (ab42018, Abcam), mouse anti-MCPIP (sc-515275, Santa Cruz). The following secondary antibodies conjugated to infrared (IR) fluorescing dyes were obtained from LI-COR: goat anti-rabbit antibody IR800CW, goat anti-mouse antibody IR680, and goat anti-mouse antibody IR800CW. Western blots were quantified using LI-COR Image Studio software. Results are presented as the MCPIP1, Dicer or TRBP expression levels normalized to actin levels, relative to negative controls. Western blot signals from the labeled secondary antibodies were normalized using the median background signal around the individual protein band. This protein signal (for MCPIP1, Dicer, or TRBP) was then divided by the signal in the same lane for beta-actin. This ratio for experimental samples was then divided by the ratio for appropriate control (e.g., mock infected, negative control miRNA).

### Co-immunoprecipitations (Co-IPs)

MC116 and MC116.219 were lysed with cold Pierce IP lysis buffer (Thermo Fisher Scientific) containing Halt protease and phosphatase inhibitor cocktail (Thermo Fisher Scientific) and incubated on ice for 30 min. Lysates were centrifuged for 15 min at 13,000 rpm at 4°C. Cell supernatant was removed and pre-cleared by adding 5 ul of washed Pierce Protein G Magnetic beads (Thermo Fisher Scientific) and rotated at 4°C for 1.5 h. The protein concentration of pre-cleared IP lysates was then determined using a Quick Start Bradford assay (Thermo Fisher Scientific). Equal amounts of protein lysates (1 ug) were used for each co-IP. Lysates were immunoprecipitated using 1 ug of mouse anti-TRBP (ab129325, Abcam), mouse anti-Dicer (ab14601, Abcam) or normal mouse IgG (sc-2025, Santa Cruz) at 4°C overnight. Pre-washed Pierce Protein G Magnetic beads (Thermo Fisher Scientific) were then added to the IPs and rotated at 4°C for 2 hrs. IP samples were resolved by SDS-PAGE along with a 5% input control, and immunoblotted with anti-Dicer or anti Ago2 (2897, Cell Signaling) antibodies.

### Statistical Analysis

At least three biological replicates were used for each analysis. Mean values and SDs were plotted using Prism (Graphpad). ANOVA as well as paired and unpaired Student’s *t* tests were used to analyze data for statistically significant differences as indicated in the legends. Values of *p < 0.05, and **p < 0.01 were regarded as statistically significant. Individual quantitative observations are included in S1 Data (main figures) and S2 Data (supporting figures).

S1 Table contains pre-miRNA sequences for in vitro cleavage assays. S2 Table contains the locations of the KSHV miRNA binding sites within the MCPIP1 3’ UTR. S3 Table contains cloning primers and gBlocks for the MCPIP1 reporter plasmids. S4 Table contains all primer probe sets (Applied Biosystems) and SYBR green qPCR primers.

## Supporting Information

S1 Fig Recombinant MCPIP1 proteins used in cleavage assays. Coomassie gel staining of in vitro translated and E. coli expressed WT MCPIP1 and D141N recombinant proteins that were used for in vitro cleavage assays.
(EPS)

S2 Fig Expression analysis from ectopic MCPIP1 expression or siRNA assays. (A) RNA samples used in Fig 2B and 2F were assessed for relative expression of MCPIP1 (WT vs. MCPIP D141N). Results are shown relative to β-actin and normalized to the 293s transfected with MCPIP1 WT with an empty vector control, N = 3–4. (B) BCBL-1 cells were nucleofected with siRNA to MCPIP1 and total RNA was harvested 48 hpt and reverse transcribed to cDNA. Expression of selected mature KSHV miRNAs was assessed using qPCR and results are shown relative to RNU48 and the siNeg control, N = 3. (C) RNA samples from Fig 2H were assessed for relative expression of MCPIP1. Results are shown relative to β-actin and normalized to the siNeg control, N = 6. For all graphs, results are shown as mean ± SD. Significance was assessed using a Student’s t test, *p ≤ 0.05, **p ≤ 0.01. Numerical data can be found in S2 Data.
(EPS)

S3 Fig Expression analysis of uninfected and infected cells. (A) HUVECs were infected with KSHV. Seven days post infection (dpi) total RNA was harvested and MCPIP1 expression was measured using qPCR. Results are shown relative to β-actin and normalized to the uninfected control, N = 12. (B) HUVECs were infected with KSHV as in A and Dicer expression was measured using qPCR, N = 11. (C) Protein lysates were isolated from 7 dpi infected HUVECs and Dicer expression was measured by Western blot analysis, N = 8. (D) Expression levels of TARBP1 and TARBP2 were measured using qPCR as described in A, N = 5. (E) Total RNA from MC116 (uninfected) and MC116.219 (KSHV infected) cells was analyzed for MCPIP1 expression using qPCR as in A, N = 4. (F) Protein lysates from MC116 and MC116.219 were used to determine expression of Dicer and TRBP following KSHV infection, N = 4. Numerical data can be found in S2 Data.
(EPS)

S4 Fig Expression analysis from miRNA, infection, and siRNA assays. (A) Dicer RNA expression was determined using qPCR from HUVECs transfected with individual KSHV miRNA mimics. Results are shown relative to β-actin and normalized to the Neg control, N = 3–8. (B) HUVECs were infected with KSHV. Seven days post infection (dpi) total RNA was harvested and AUF1 expression was measured using qPCR, N = 4. (C) HUVECs were transfected with siRNA and RNA was isolated 48 hpt and used for qPCR to quantify the expression of Dicer. Results are shown relative to β-actin and normalized to the siNeg control, N = 4. (D) HUVECs were transfected with siRNA and whole cell protein lysates were collected 48 hpt and measured using western blot analysis, N = 3–4. Results are shown relative to β-actin and normalized to the siNeg control. (E) A549 cells were transfected with siRNA and RNA was isolated 48 hpt and used for qPCR. MCPIP1 and Dicer expression was assessed using taqman probes relative to β-actin. Fold change was calculated and normalized to the siNeg control, N = 3–4. (F) HUVECs were transfected with siRNA and RNA was isolated 48 hpt and used for qPCR to quantify the expression of MCPIP1. Results are shown relative to β-actin and normalized to the siNeg control, N = 4. (G) WT and MCPIP1 -/- MEFs were transfected with 50 ng of a MCPIP1 expression vector or an EGFP negative control vector and RNA was isolated 48 hpt. RNA was DNase treated and analyzed for MCPIP1 expression using qPCR. Results are shown relative to β-actin and normalized to the WT control, N = 4. For all graphs, results are shown as mean ± SD. Significance was assessed using a Student’s t test, *p ≤ 0.05, **p ≤ 0.01. Numerical data can be found in S2 Data.
(EPS)

S5 Fig IL-1b activation of MCPIP1 expression and expression of viral genes upon induced MCPIP1 expression. (A) BCBL-1 cells were nucleofected with siRNA to MCPIP1 followed by stimulation with 15 ng/ul IL-1β for 24 hrs. RNA was isolated and MCPIP1 mRNA expression was measured using qPCR. Results are shown relative to β-actin and normalized to the siNeg, untreated control, N = 3. (B) 293 Doxycycline inducible GFP-MCPIP1 cells were induced with doxycycline (10 ng/ul) for 24 hours to express MCPIP1 followed by KSHV infection. Doxycycline was replaced after infection and RNA was isolated 3 dpi. MCPIP1 mRNA expression was measured using qPCR analysis. Results are shown relative to β-actin and normalized to the mock infected, untreated control. Significance was assessed using a Student’s t test, *p ≤0.05, **p ≤0.01, N = 4–5. (C) 293 Doxycycline inducible GFP-MCPIP1 cells were induced with doxycycline (10 ng/ul) for 24 hours to express MCPIP1 followed by KSHV infection (as in A). KSHV markers for latent infection (LANA and vFLIP) and lytic infection (orf69, K14 and orf57) were evaluated. Results are shown relative to GAPDH and normalized to doxycycline negative, KSHV infected cells. For all graphs, results are shown as mean ± SD. Significance was assessed using a Student’s t test, *p ≤0.05, **p ≤0.01, N = 3–5. Numerical data can be found in S2 Data.
(EPS)

S1 Table Pre-miRNA sequences used for in vitro cleavage assays. Table contains pre-miRNA sequences for in vitro cleavage assays.
(XLSX)

S2 Table KSHV miRNA target sites within the MCPIP1 3'UTR. Table contains the locations of the KSHV miRNA binding sites within the MCPIP1 3’ UTR.
(XLSX)

S3 Table Primers used for cloning and mutagenesis. Table contains cloning primers and gBlocks for the MCPIP1 reporter plasmids.
(XLSX)

S4 Table Primers used for expression analysis. Table contains all primer probe sets (Applied Biosystems) and SYBR green qPCR primers.
(XLSX)

S1 Data Quantitative information for Fig 2 through Fig 7. Tables with numerical data for Figs 2, 3, 4, 5, 6 and 7.
(XLSX)

S2 Data Quantitative information for S2 Fig through S5 Fig. Tables with numerical data for S2, S3, S4 and S5 Figs.
(XLSX)

## Abbreviations

AUF1: AU-binding factor-1
DEN: dengue virus
EGFP: Enhanced Green Fluorescent Protein
HCV: hepatitis C virus
hnRNP D: heterogeneous nuclear ribonucleoprotein D
HUVEC: primary human umbilical vein endothelial cell
IL-1β: interleukin-1β
JEV: Japanese encephalitis virus
KS: Kaposi’s sarcoma
KSHV: Kaposi’s sarcoma-associated herpesvirus
LNA: locked nucleic acid inhibitor
MCD: multicentric Castleman’s disease
MCP-1: monocyte chemoattractant protein-1
MCPIP1: MCP-1-induced protein-1
MEF: mouse embryonic fibroblast
miRISC: miRNA-induced silencing complex
miRLC: miRNA RISC loading complex
miRNA: microRNA
PEL: primary effusion lymphoma
pre-miRNA: precursor miRNA
pri-miRNA: primary microRNA
RISC: RNA-induced silencing complex
TARBP2: HIV-1 TAR-RNA Binding Protein
TNFα: tumor necrosis factor α
TWEAK: tumor necrosis factor-like weak inducer of apoptosis
